# Adverse childhood experiences and food insecurity in emerging adulthood: findings from the EAT 2010–2018 study

**DOI:** 10.1017/S1368980023001349

**Published:** 2023-11

**Authors:** Nicole Larson, Susan M Mason, Meg Bruening, Melissa N Laska, Vivienne M Hazzard, Dianne Neumark-Sztainer

**Affiliations:** 1 Division of Epidemiology and Community Health, School of Public Health, University of Minnesota, Suite 300, 1300 South Second Street, Minneapolis, MN 55454, USA; 2 Department of Nutritional Sciences, College of Health and Human Development, Penn State, 110 Chandlee Lab, University Park, PA 16802, USA

**Keywords:** Food insecurity, Emerging adulthood, Adverse childhood experiences, Socio-economic status

## Abstract

**Objective:**

Low childhood socio-economic status (SES) and adverse childhood experiences (ACE) are associated with poor health outcomes in adulthood. Determining how ACE may be linked to food insecurity among young people from socio-economically diverse households can inform health-protective strategies. This study examined if ACE are associated with food insecurity during the transition to adulthood and investigated prevalence differences across SES strata.

**Setting:**

Participants were recruited from twenty secondary schools in Minneapolis-St. Paul, Minnesota.

**Participants:**

The analytic sample (*n* 1518) completed classroom surveys in 2009–2010 (mean age = 14·5 years) and follow-up surveys in 2017–2018 (mean age = 22·0 years).

**Design:**

Past-year food insecurity was reported at both time points, and ACE were reported at follow-up. Logistic regression models were used to estimate emerging adult food insecurity prevalence by ACE exposure; models were stratified by childhood SES (low, middle and high).

**Results:**

The adjusted prevalence of food insecurity was 45·3 % among emerging adults who reported three or more ACE compared with 23·6 % among those with one or two ACE and 15·5 % among those with no ACE (*P* < 0·001). All forms of ACE were related to an elevated prevalence of food insecurity in emerging adulthood. ACE–food insecurity associations were strongest for emerging adults from lower and middle SES households. Among emerging adults from low SES households, childhood experiences of emotional abuse and substance use by a household member were associated with the largest prevalence differences in food insecurity.

**Conclusions:**

Findings suggest a need for trauma-informed services within food assistance programs to better serve individuals with a history of ACE.

Adverse childhood experiences (ACE), including abuse and household dysfunction, are prevalent public health problems. More than 60 % of U.S. adults report one or more ACE and 25 % report three or more ACE^(1)^. The trauma and toxic stress associated with ACE can alter brain development and negatively impact mental and physical health across the life course, including increased risk for early-onset chronic disease in young adulthood^(2–4)^. Associations of ACE with later life disease may stem from biological, behavioural and social pathways^(3)^. For example, research has identified a link between ACE and early-stage immune responses (e.g. metabolically induced inflammation) that have direct, negative consequences for biological outcomes^(3)^. ACE may also influence health outcomes through psychosocial determinants (e.g. isolation) and lifestyle behaviours (e.g. energy-dense food consumption)^(3)^. With regard to social mechanisms, there is growing evidence that links ACE to later poor educational outcomes, low wages and food insecurity^(5,6)^. Accordingly, the primary prevention of ACE is a public health priority; in cases where ACE cannot be prevented, it is also crucial that health programs and policies are designed to promote the well-being of persons who have experienced early-life adversities.

Programs and policies that consistently increase access to adequate, nutrient-dense food are critical to maintaining the well-being of young people throughout childhood and the transition to adulthood. The transition to adulthood, a life stage often termed ‘emerging adulthood’ (18–29 years), is a period of vulnerability for experiencing food and nutrition insecurity and its potential impacts on health outcomes such as poor diet, disordered eating, elevated blood pressure and prediabetes^(7,8)^. The prevalence of food insecurity was particularly high among emerging adults during the initial months of the COVID-19 pandemic with one recent study reporting close to 30 % of young people had inadequate resources to purchase food in the past year^(9)^. Providing emerging adult populations with the supports needed to ensure adequate food and nutrient intake is important for the promotion of well-being, including cardiometabolic health and positive reproductive outcomes. The existing literature has demonstrated a relationship between ACE and increased risk for food insecurity; however, few studies have examined the relationship among population-based samples of emerging adults and existing studies have not had the capacity to address the potential role of childhood socio-economic status (SES)^(6,10)^ in this relationship. There is a lack of research addressing how different forms of ACE may be related to risk for food insecurity and the potential for household SES in childhood to confound or modify observed relationships.

Multiple pathways may contribute to observed linkages between ACE and future food insecurity. Cumulative disadvantage theory posits that adversities tend to cluster and mount up over the life course^(11,12)^. In line with this theory, there is evidence that individuals with ACE have greater social adversities in adulthood. Many of the psychological and social outcomes associated with ACE (e.g. below average school performance and educational achievement, low wages and depressive symptoms) may contribute to increased risk for future food insecurity^(13–15)^. Low childhood SES is another adversity that is related to both ACE and to future adult SES and food insecurity^(15,16)^. Young people from households with lower incomes are more than twice as likely to be exposed to ACE compared with their peers from households with higher incomes^(15)^. It is possible that some portion of the observed relationship between ACE and future food insecurity may reflect confounding by childhood SES. However, it is further possible that the combination of low childhood SES and ACE may create particularly heightened vulnerability to later social challenges. More specifically, the association between ACE and food insecurity may be exacerbated in contexts with fewer potential buffering resources.

The pathways contributing to food insecurity may differ across forms of ACE given their different impacts on emotion regulation development, executive functioning and stress sensitisation^(17)^. For example, one prior study of U.S. adults observed that health risks associated with some ACE (i.e. exposure to domestic violence, parental divorces and residing with a parent who was incarcerated) were nearly entirely explained by adult SES conditions, whereas SES conditions explained only a small portion of observed associations between other forms of ACE (i.e. physical, emotional and sexual abuse) and health risks^(18)^. Despite evidence linking ACE to markers of adult SES and emotional well-being, little research has explored the extent to which these factors mediate the relationship between ACE and adult experiences of food insecurity^(18)^. Likewise, few studies have examined the extent to which this association may be modified by childhood SES. It is important for programmes and services for emerging adults to be informed by evidence regarding the role of economic factors and the potential need for attending to barriers to food security that are unrelated to economic factors.

The current study will extend the evidence base by examining how ACE are associated with experiencing food insecurity during the transition to adulthood among an ethnically/racially and socio-economically diverse cohort. For this study, ACE are examined with a focus on physical, sexual and emotional abuse; incarceration of a household member; substance abuse by a household member and having a household member with a mental health problem. The aims are to (1) examine how ACE, including individual types and cumulative number, are associated with food insecurity in emerging adulthood and (2) examine observed associations by household SES in adolescence. Figure 1 illustrates the conceptual model guiding the approach. It is hypothesised that the prevalence of food insecurity will be elevated among emerging adults with a history of any form of ACE and observed prevalence differences will be largest when comparing those with multiple ACE to those with no ACE. It is also hypothesised that household SES in adolescence and markers of emotional well-being will not fully explain prevalence differences in food insecurity by ACE exposure.

**Fig. 1:**
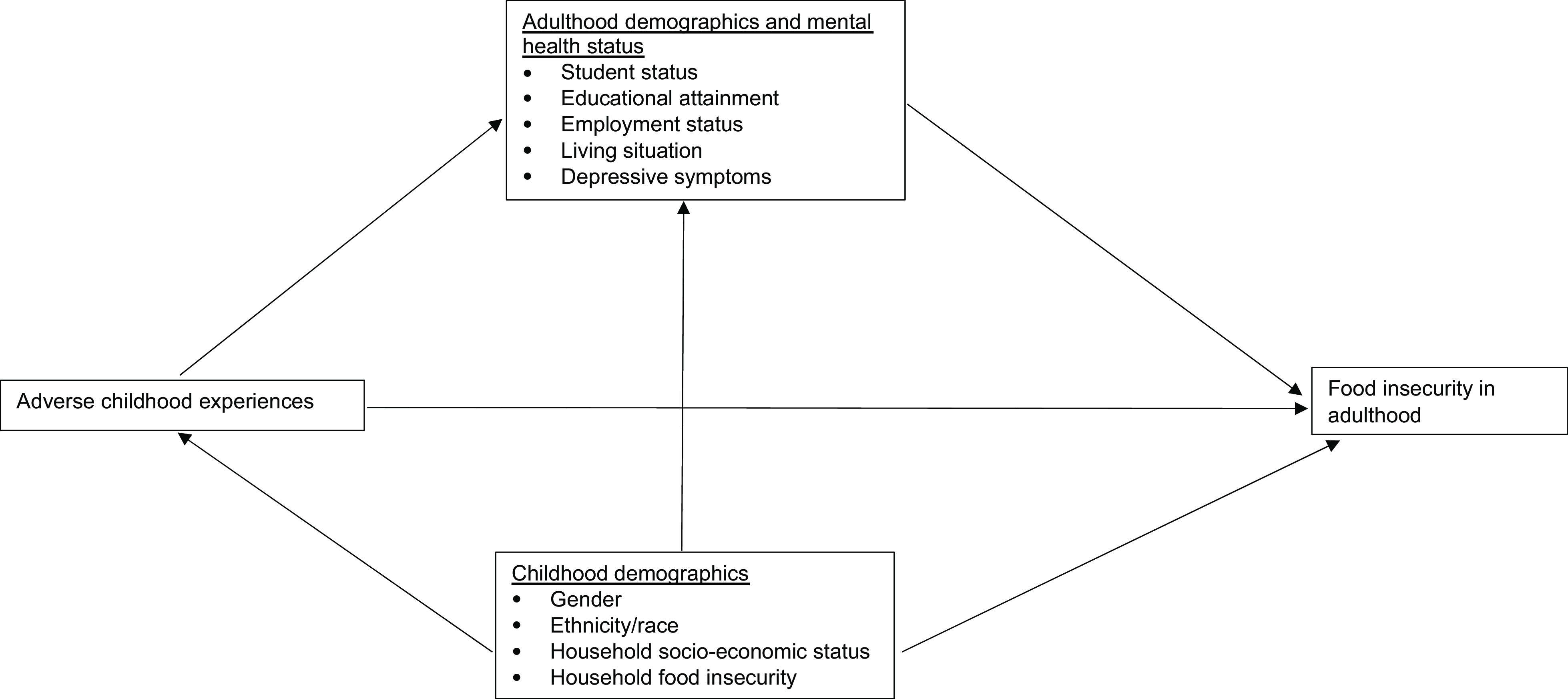
Conceptual model guiding analysis of the associations between adverse childhood experiences, childhood socio-economic status and food insecurity in emerging adulthood among participants in the EAT 2010–2018 (Eating and Activity over Time) longitudinal study

## Methods

### Study design and population

EAT 2010–2018 (Eating and Activity over Time) is a population-based, longitudinal study of weight-related health behaviours and associated factors. The analytic sample included 878 female participants, 629 male participants and eleven participants identifying with another gender identity. The EAT 2010 study recruited adolescents during the 2009–2010 academic year to complete surveys in middle and senior high school classrooms at twenty urban public schools in Minneapolis-St. Paul, Minnesota^(19)^. Schools were selected based on students’ demographic characteristics as an important goal of the study was to learn about the weight-related health of ethnically/racially and socio-economically diverse adolescents. Participants in EAT 2010 were asked to respond to the follow-up EAT 2018 survey in 2017–2018^(20)^. EAT 2018 was designed to examine changes in weight-related behaviours (e.g. eating and physical activity patterns) as participants progressed through adolescence and into young adulthood. Invitations to the online EAT 2018 survey were mailed to all EAT 2010 participants from whom contact information was available (*n* 2383 of 2793). Participants were provided a financial incentive following survey completion. The University of Minnesota Institutional Review Board Human Subjects Committee approved all protocols.

The diverse sample of 1568 participants who completed surveys at both time points represents 65·8 % of original participants for whom contact information was available at EAT 2018. There were 410 original participants who were lost to follow-up, primarily due to missing contact information at EAT 2010 or no address found for EAT 2018. Another fifty participants responded to the EAT 2018 survey, but were excluded from the current analysis because they did not respond to the survey measures of food insecurity. As attrition did not occur completely at random, inverse probability weighting (IPW) was used to account for missing data^(21)^. IPW minimises response bias due to missing data and allows for extrapolation back to the original EAT 2010 school-based sample. There were no statistically significant demographic differences between the EAT 2010 sample of adolescents and the weighted EAT 2018 survey respondent sample. Online Supplementary Table 1 provides a description of the analytic sample, including 340 participants who had been food insecure and 1178 participants who had been food secure in the past year prior to completing the EAT 2018 survey.

### Survey development and measures

The EAT 2010 and EAT 2018 surveys were developed by a team of experts in the domains of nutrition, physical activity, adolescent development, body image and family relations^(19,20,22)^. The EAT 2018 survey was based on EAT 2010 and other surveys of weight-related health^(19)^. Pretesting and pilot testing were conducted for both surveys. For EAT 2010, the survey was pretested by fifty-six adolescents with diverse backgrounds and pilot tested with a separate sample of 129 middle school and high school students to examine the test-retest reliability of measures over a 1-week period. Additionally, for EAT 2018, focus groups (*n* 29) were conducted to pretest the survey and the test-retest reliability of measures was examined using data from 112 participants who completed the survey twice over 3 weeks.

All survey measures are described along with details of item test-retest reliability in Table 1. Adolescent food security status, household SES, gender and ethnicity/race were assessed on the EAT 2010 survey^(19,23,24)^. The EAT 2018 survey was the source of all other measures, including emerging adult food security status, ACE, depressive symptoms, living situation, educational attainment, employment status and student status^(25–34)^.

**Table 1:** Description of survey measures

Construct	Survey measure	Response categories	Test-retest reliability and validity
Traumatic experiences			
Adverse childhood experiences (ACE)	Seven items that were modified from the Adverse Childhood Experiences scale^(28,31)^ and the Childhood Trauma Questionnaire^(29)^. Participants were asked if any of the following had ever happened prior to their 18th birthday: *a household member was depressed, mentally ill, or attempted suicide*; *you lived with someone who was a problem drinker or alcoholic, who used street drugs, or who abused prescription drugs; a household member went to prison;* and *someone [in/outside] your family touched you in a sexual way against your wishes or forced you to touch them in a sexual way.* Participants were also asked how often, prior to their 18th birthday, an adult in their family had *said hurtful or insulting things to me* and *hit me so hard it left me with bruises or marks.*	*Yes/No* and, for reported of frequency of emotional and physical abuse, *Never*, *rarely*, *sometimes*, *often* and *very often*	Test-retest reliability was assessed for the sum of the seven items (*r* = 0·71) and test-retest agreement for each form of ACE: mental health problem (81 %), substance abuse (87 %), incarceration (94 %), sexual abuse (92 %), physical abuse (90 %) and emotional abuse (87 %). A variable summarising the number of forms of ACE was derived with the categories none, 1–2 and 3+ forms. The test-retest agreement for this summary variable was 83 %.
Food insecurity experiences			
Emerging adult food insufficiency	Two items from the short form of the U.S. Household Food Security Survey Module^(25)^: • In the last 12 months, did you ever eat less than you felt you should because there wasn’t enough money for food? • In the last 12 months, were you ever hungry but didn’t eat because there was not enough money for food?	*Yes/No/I don’t know*.	Test-retest agreement was high for the statement about eating less (83 %) and the statement about hunger (79 %). If participants responded ‘yes’ to both questions, then participants were categorised as food insufficient.
Adolescent food insecurity	Items were modified from the U.S. Household Food Security Survey Module^(32,33)^: *• How often during the last 12 months have you been hungry because your family couldn’t afford more food?* *• Which of these statements best describes the food eaten in your home in the last 12 months?*	Hunger: *Almost every month, some months but not every month, only one or two months, I have not been hungry for this reason* Food in your home: *Often we don’t have enough to eat, sometimes we don’t have enough to eat, we have enough to eat but not always the kinds of food we want, we always have enough to eat and the kinds of food we want*	If participants indicated they had ever been hungry and experienced any compromise to food adequacy, then they were categorised as food insecure. Test-retest agreement for food security status was 96 %.
Depressive symptoms and personal characteristics			
Depressive symptoms	Kandel and Davies’ six-item scale^(34)^ was used to assess symptoms of depression, *including feeling hopeless about the future; feeling too tired to do things; having trouble going to sleep or staying asleep; feeling unhappy, sad or depressed; feeling nervous or tense; worrying too much about things*	*Not at all, Somewhat, Very much*	Items were summed and the scale was found to be reliable (Cronbach’s alpha = 0·88, test-retest reliability *r* = 0·71.)
Employment status	*Which of the following best describes your current work situation?*	*Working full-time, working part-time, stay at home caregiver, currently unemployed, but actively seeking work, not working for pay, other*	Test-retest agreement was strong (83 %).
Student status	*Which of the following best describes your student status (for the majority of the past year)?*	Seven response options ranged from *not a student* to *graduate student part-time or full-time.* Participants were categorised as not a student; a full-time student in high school or postsecondary enrollment option or a college/university student.	Test-retest agreement was strong (92 %).
Educational attainment	*What is the highest level of education that you have completed?*	Response options ranged from *middle school or junior high* to *graduate or professional degree (MS, MBA, MD, PhD, etc).*	Test-retest agreement was strong (96 %).
Living situation	*During the past year, with whom did you live the majority of the time?*	Participants were asked to select all that apply from a list of eight options (e.g. *I live alone; my child(ren), including any step-children or adopted children*). Based on responses, participants were categorised with regard to whether or not they were living with a parent and/or a child of their own.	Test-retest agreement was strong (100 %).
Demographic characteristics	Self-report of gender, ethnicity/race and markers of household socio-economic status (SES) in childhood. Ethnicity/race and SES were based on measures included on the original school-based survey. The prime determinant of SES was the higher educational level of either parent, with adjustments made for student eligibility for free/reduced price school meals, family public assistance receipt and parent employment status.		Test-retest agreement was strong for ethnicity/race (98–100 %) and the measure of SES (*r* = 0·90).

### Statistical analysis

The distribution of food insecurity among emerging adults who have experienced different forms of ACE was examined by using binomial regression models and marginal standardisation to calculate predicted prevalences and 95 % CI^(35,36)^. Separate regression models were examined for each form of ACE and for a categorical, derived variable summarising the total number of ACE reported. Regression models were examined first with adjustment only for adolescent characteristics (gender, ethnicity/race, household SES and food insecurity in model 1) and then with additional adjustment for emerging adult characteristics (student status, educational attainment, employment status, living situation and depressive symptoms in model 2) that may operate as mediators of the ACE-food insecurity association. SES-stratified regression models were likewise examined separately for each form of ACE and with adjustment for only adolescent characteristics (model 1) and then with additional adjustment for emerging adult characteristics (model 2). Regression models that further were mutually adjusted for each form of ACE were also examined first with adjustment only for adolescent characteristics (model 1) and with adjustment for both adolescent and emerging adult characteristics (model 2).

Analyses were conducted using the Statistical Analysis System (version 9.4, 2015, SAS Institute Inc.) and, as described above, used IPW to account for missing data^(21)^. All *P* values were recorded to three decimal places in the tables and the results are presented in a manner that emphasises patterns and the magnitude of observed associations.

## Results

### Associations between adverse childhood experiences and food insecurity in emerging adulthood

Experiencing each form of ACE was related to a higher prevalence of emerging adult food insecurity, relative to not having that ACE, in models adjusted for adolescent characteristics (Table 2a, Model 1). Emotional abuse was linked to the biggest difference in food insecurity prevalence, with those experiencing emotional abuse having a 46·7 % prevalence of food insecurity compared with a 19·5 % prevalence among those without this ACE (Table 2a, Model 1). Other forms of ACE were somewhat less strongly linked to food insecurity; prevalences ranged from 35·5 % for those with sexual abuse histories (relative to 20·8 % in those without) to 37·9 % for those with exposure to substance abuse by a household member (relative to 18·4 % in those without). Results based on models with adjustment for both adolescent and emerging adult characteristics also showed that experiencing each form of ACE was related to a higher prevalence of emerging adult food insecurity; however, prevalence differences were attenuated relative to model 1 (Table 2a, model 2).

**Table 2a:** Prevalence (95 % CI) of past-year food insecurity in emerging adulthood by history of ever or never having a form of adverse childhood experience (ACE)*

	Model 1†					Model 2‡				
	Ever for ACE: % food insecure	95 % CI	Never for ACE: % food insecure	95 % CI	*P* value for ACE	Ever for ACE: % food insecure	95 % CI	Never for ACE: % food insecure	95 % CI	*P* value for ACE
Form of ACE										
Sexual abuse	35·5	28·8, 42·2	20·8	18·5, 23·0	< 0·001	28·1	22·3, 33·8	22·1	19·9, 24·3	0·048
Physical abuse	36·0	30·1, 41·9	20·1	17·9, 22·4	< 0·001	27·9	22·8, 32·9	22·0	19·7, 24·2	0·030
Emotional abuse	46·7	39·4, 54·0	19·5	17·3, 21·6	< 0·001	35·8	29·0, 42·6	21·0	18·8, 23·1	< 0·001
Incarceration of household member	40·7	33·3, 48·1	20·6	18·4, 22·8	< 0·001	31·8	25·4, 38·3	21·9	19·8, 24·1	0·002
Substance abuse by household member	37·9	32·6, 43·2	18·4	16·1, 20·7	< 0·001	29·7	25·1, 34·4	20·7	18·3, 23·1	< 0·001
Mental health problem of household member	37·8	33·0, 42·7	17·6	15·3, 19·8	< 0·001	30·3	25·9, 34·7	19·9	17·5, 22·3	< 0·001

* Marginal standardisation was used to calculate predicted prevalences. Prevalence values are weighted to reflect the probability of responding to the follow-up EAT 2018 survey. In each column of prevalences, cells that share a superscript letter do not differ (*P* > 0·050).

† Model 1 includes gender, ethnicity/race, childhood socio-economic status and history of childhood food insecurity.

‡ Model 2 includes all of the variables in model 1 along with emerging adult employment status, student status, educational attainment, living situation and depressive symptoms.

Predicted prevalences of food insecurity were also estimated using regression models that were mutually adjusted for each form of ACE (Table 2b). These models similarly showed that emotional abuse was linked to the biggest difference in food insecurity prevalence. After adjustment for both adolescent characteristics and all other forms of ACE, those experiencing emotional abuse had a 37·4 % prevalence of food insecurity compared with a 20·3 % prevalence among those without this ACE (Table 2b, Model 1). Other forms of ACE were somewhat less strongly linked to food insecurity; prevalences ranged from 24·2 % for those with sexual abuse histories (relative to 22·5 % in those without) to 28·7 % for those with a household member having a mental health problem (relative to 20·3 % in those without). Results based on models with adjustment for multiple forms of ACE and both adolescent and emerging adult characteristics similarly showed that emotional abuse was linked to the biggest difference in food insecurity prevalence (Table 2b, Model 2).

**Table 2b:** Mutually adjusted prevalences (95 % CI) of past-year food insecurity in emerging adulthood by history of ever or never having a form of adverse childhood experience (ACE)*

	Model 1†					Model 2‡				
	Ever for ACE: % food insecure	95 % CI	Never for ACE: % food insecure	95 % CI	*P* value for ACE	Ever for ACE: % food insecure	95 % CI	Never for ACE: % food insecure	95 % CI	*P* value for ACE
Form of ACE										
Sexual abuse	24·2	18·4, 30·0	22·5	20·2, 24·8	0·598	23·4	18·0, 28·9	23·0	20·7, 25·3	0·887
Physical abuse	24·5	19·2, 29·8	22·4	20·0, 24·8	0·481	23·0	18·1, 27·9	23·1	20·7, 25·4	0·980
Emotional abuse	37·4	29·9, 45·0	20·3	18·0, 22·6	< 0·001	33·2	26·2, 40·1	21·2	19·0, 23·4	< 0·001
Incarceration of household member	27·4	20·7, 34·1	22·0	19·7, 24·3	0·125	26·3	20·0, 32·6	22·5	20·2, 24·7	0·268
Substance abuse by household member	28·5	23·2, 33·9	20·8	18·2, 23·3	0·011	26·0	21·1, 30·8	21·9	19·4, 24·5	0·162
Mental health problem of household member	28·7	23·8, 33·6	20·3	17·6, 22·9	0·003	26·3	21·8, 30·8	21·5	18·9, 24·2	0·086

* Marginal standardisation was used to calculate predicted prevalences. Prevalence values are weighted to reflect the probability of responding to the follow-up EAT 2018 survey. In each column of prevalences, cells that share a superscript letter do not differ (*P* > 0·050).

† Model 1 includes gender, ethnicity/race, childhood socio-economic status and history of childhood food insecurity.

‡ Model 2 includes all of the variables in model 1 along with emerging adult employment status, student status, educational attainment, living situation and depressive symptoms.

When examined as a cumulative score, the number of ACE was also significantly related to food insecurity prevalence in the model adjusted for adolescent characteristics and the model adjusted for both adolescent and emerging adult characteristics (Table 2c). The model including adolescent characteristics showed the adjusted prevalence of food insecurity was 45·3 % among emerging adults who reported three or more ACE compared with 23·6 % among those with one or two ACE, and 15·5 % among those with no ACE (model 1). The model including adolescent and emerging adult characteristics showed the adjusted prevalence of food insecurity was 34·8 % among emerging adults who reported three or more ACE compared with 21·8 % among those with one or two ACE and 19·2 % among those with no ACE (model 2).

**Table 2c:** Mutually adjusted prevalences (95 % CI) of past-year food insecurity in emerging adulthood by number of adverse childhood experiences (ACE)*

	Model 1†				Model 2‡		
	Adjusted prevalences	95 % CI	*P* value for count		Adjusted prevalences	95 % CI	*P* value for count
Count of ACE			< 0·001				< 0·001
None	15·5	13·0, 18·0^a^			19·2	16·3, 22·2^a^	
1–2	23·6	19·5, 27·7^b^			21·8	18·1, 25·6^a^	
3+	45·3	39·1, 51·6^c^			34·8	29·0, 40·6^b^	

* Marginal standardisation was used to calculate predicted prevalences. Prevalence values are weighted to reflect the probability of responding to the follow-up EAT 2018 survey. In each column of prevalences, cells that share a superscript letter do not differ (*P* > 0·050).

† Model 1 includes gender, ethnicity/race, childhood socio-economic status and history of childhood food insecurity.

‡ Model 2 includes all of the variables in model 1 along with emerging adult employment status, student status, educational attainment, living situation and depressive symptoms.

### Modification of associations between adverse childhood experiences and food insecurity by household socio-economic status

The SES-stratified predicted prevalences of food insecurity were estimated for groups of emerging adults who indicated ever experiencing a specific form of adversity and compared with those with no history of the specific form of ACE (Table 3). Among emerging adults from *low SES households*, models adjusted for adolescent characteristics suggested elevated food insecurity among those with most types of ACE; the exception was for those with a history of sexual abuse the prevalence of food insecurity (37·4 %) was similar to the prevalence for those who did not experience sexual abuse (27·2 %), with overlapping confidence intervals. Physical abuse was associated with a modest increase in food insecurity (38·8 % *v*. 25·6 %), a prevalence difference of 13 %, whereas emotional abuse (50·3 % *v*. 24·7 %) and substance abuse by a household member (48·0 % *v*. 22·3 %) were associated with the largest prevalence differences of approximately 26 %. In *middle SES households*, prevalence differences in food insecurity ranged from 18 % for those with *v*. without exposure to substance abuse by a household member (34·3 % *v*. 16·0 %) to nearly 24 % for those with *v*. without a history of emotional abuse (40·8 % *v*. 17·0 %). Among emerging adults from *high SES households*, prevalence differences were attenuated, with only emotional abuse and a household member having a mental health problem being associated with a difference of more than 10 %. The highest predicted prevalence of food insecurity among emerging adults from high SES households was 56·6 % for those who reported emotional abuse compared with 14·9 % among those who did not report emotional abuse.

**Table 3:** Adjusted prevalence of past-year food insecurity in emerging adulthood by history of ever or never having an adverse childhood experience (ACE) and low, middle or upper socio-economic status (SES)*,†,‡,§

	Low SES					Middle SES					High SES				
	Ever for ACE: % food insecure	95 % CI	Never for ACE: % food insecure	95 % CI	*P* value for ACE	Ever for ACE: % food insecure	95 % CI	Never for ACE: % food insecure	95 % CI	*P* value for ACE	Ever for ACE: % food insecure	95 % CI	Never for ACE: % food insecure	95 % CI	*P* value for ACE
Form of Adverse Childhood Experience															
Sexual abuse	37·2	25·8, 48·5	27·2	23·3, 31·1	0·090	39·3	28·7, 49·8	16·5	13·2, 19·8	< 0·001	22·4	9·6, 35·3	17·1	12·7, 21·6	0·421
Physical abuse	38·8	30·2, 47·4	25·6	21·6, 29·6	0·004	37·2	27·7, 46·7	16·8	13·5, 20·2	< 0·001	26·8	11·8, 41·8	16·8	12·4, 21·1	0·160
Emotional abuse	50·3	39·1, 61·5	24·7	20·9, 28·5	< 0·001	40·8	29·8, 51·7	17·0	13·8, 20·3	< 0·001	56·6	35·8, 77·5	14·9	10·9, 19·0	< 0·001
Incarceration of household member	49·2	37·6, 60·7	25·5	21·7, 29·3	< 0·001	38·0	26·6, 49·5	17·8	14·5, 21·2	< 0·001	25·8	9·9, 41·7	17·0	12·7, 21·3	0·244
Substance abuse by household member	48·0	39·2, 56·7	22·3	18·3, 26·2	< 0·001	34·3	26·0, 42·5	16·0	12·6, 19·5	< 0·001	23·3	12·6, 34·0	16·2	11·7, 20·8	0·200
Mental health problem of household member	44·6	36·0, 53·3	23·4	19·4, 27·3	< 0·001	35·2	27·7, 42·6	14·4	11·0, 17·8	< 0·001	28·3	19·3, 37·4	13·6	9·0, 18·1	0·003

* The primary determinant of SES was parental educational level, defined by the higher level of either parent. Additional measures of income and employment were used as part of an algorithm to reduce the impact of missing data and to prevent misclassification in ranking SES (range: 1–5). Low SES was defined as rank 1, middle SES as rank 2–3 and high SES as rank 4–5.

† The model includes gender, ethnicity/race and history of childhood food insecurity.

‡ Marginal standardisation was used to calculate predicted prevalences. Prevalence values are weighted to reflect the probability of responding to the follow-up EAT 2018 survey.

§ There were young people of low (L), middle (M) and high (H) SES backgrounds who experienced each form of ACE. For each form of ACE, the counts are reported here: sexual abuse (L = 75, M = 94, H = 53), physical abuse (L = 110, M = 95, H = 34), emotional abuse (L = 75, M = 86, H = 25), incarceration of a household member (L = 64, M = 69, H = 29), substance abuse by a household member (L = 119, M = 139, H = 66) and mental health problem of a household member (L = 119, M = 168, H = 111).

## Discussion

This study investigated relationships between ACE and experiences of food insecurity in a population-based sample of emerging adults. Results of this study extend existing research on associations between ACE and childhood food insecurity by showing that all forms of ACE were related to an elevated prevalence of food insecurity in emerging adulthood, and these relationships were similar for young people from lower and middle SES households. The adverse experience of emotional abuse was most robustly linked to emerging adult food insecurity, with close to half of those who experienced emotional abuse indicating they were food insecure in the past year. Additionally, the results showed there was a stepwise increase in the prevalence of food insecurity among emerging adults as the number of ACE increased. This observation of a cumulative relationship between ACE and food insecurity adds to growing evidence that those with a history of ACE may be in particular need of food assistance programs. It seems likely that the individuals with ACE histories who experience food insecurity are less likely than others to have had access to supports to address the sequelae of ACE. Therefore, it could be beneficial for food assistance programs to develop resources for reaching emerging adults with ACE histories as one potential point connecting them to resources such as free or sliding-fee mental healthcare.

The results are consistent with the literature addressing specific associations between various forms of ACE and food insecurity. Two prior studies have examined these relationships among nationally representative samples of children and young adults, and similarly observed that most forms of ACE are related to an increased prevalence of food insecurity^(10,37)^. For example, among a sample of 12 288 young adults, Testa and Jackson separately examined associations between food insecurity and the ACE of emotional abuse, physical abuse, sexual abuse, low parental warmth, physical neglect, community violence, substance abuse in the household, parental separation or divorce, suicide exposure and incarceration of a household member^(10)^. Testa and Jackson observed odds ratios for food insecurity ranging from 1·29 for community violence to 1·68 for parental incarceration in models that accounted for demographic factors and potential mediators (e.g. depressive symptoms); low parental warmth, physical neglect and suicide exposure were the only forms of ACE that were not associated with food insecurity^(10)^. The current study adds to these prior findings by demonstrating that associations between ACE and food insecurity are present even after adjusting for adolescent food insecurity as a potential confounder. Finally, by mutually adjusting for all different forms of ACE, we aimed to identify what ACE may be most important in predicting food insecurity and found a particularly strong association between childhood emotional abuse and future risk for food insecurity. Emotional abuse has been linked to higher levels of depression, but little is known about its relationship to other risk factors for experiencing food insecurity (e.g. below average school performance, perceptions of food adequacy)^(38)^.

Findings of the current study also extend what is known about the consistency of observed relationships among young people of diverse, childhood SES backgrounds. Prior research has demonstrated that both ACE and childhood food insecurity are associated with future educational attainment, but few studies have examined whether parental education moderates the relationship between ACE and food insecurity^(39)^. Higher levels of parent education, which are linked to higher parent incomes, may reduce the risk of future food insecurity in young people by increasing child educational attainment and income potential. Further, higher parent education and the household resources that often come with it may help to buffer the impacts of ACE. For the current study, household SES was primarily determined by the educational attainment of parent(s) at the time when participants were first enrolled in school classrooms. The results provide limited evidence that high household SES in childhood may buffer the relationship between some forms of early life adversities and future food insecurity in emerging adulthood. Though notably, emotional abuse and a household member having a mental health problem were strongly related to food insecurity even in high-SES households. It is also important to acknowledge there was a relatively small number of participants who experienced childhood emotional abuse and were from high SES households (*n* 25), and therefore, the CI for the adjusted prevalence of adult food insecurity among this subsample is wide (35·8–77·5 %). However, the estimated prevalence of adult food insecurity among participants who experienced emotional abuse was very similar among those from low SES households (50·3 %) as compared with those from high SES households (56·6 %). The results are in line with related research among the Avon Longitudinal Study of Parents and Children that found associations of ACE with some educational and health outcomes (e.g. depression, drug use, and smoking) were not altered by adjustment for socio-economic factors^(39)^. Additional research will be needed to clarify the role of household SES; however, the results imply that interventions should not focus solely on ACE or solely on socio-economic deprivation, as both appear to be important predictors of future food insecurity. For example, it may be informative for qualitative research to explore the degree to which emerging adults with a history of childhood emotional abuse may benefit from the resources of their parental household and the role of challenges related to executive functioning^(40)^. Similarly, future studies could help to address whether the resources of high SES households may be sufficient to overcome the potential impact of most other types of ACE on a young person’s ability to perform well in school and as an adult earn an adequate income for purchasing food.

Strengths and limitations of the current study should be considered as part of interpreting the findings. Key strengths include the large, population-based sample of sociodemographically diverse participants, the ability to adjust for adolescent food security status as a potential confounder and the broad range of variables that were used to investigate the potential influence of different types of ACE on risk for experiencing food insecurity. The diversity of participants and large sample size allowed the study to conduct analyses adjusting for gender, ethnicity/race, markers of SES and living situation. Two main limitations warrant consideration. First, both ACE and food insecurity were assessed by survey self-report and were kept brief to limit participant burden. Food insecurity associated with food access problems is most reliably assessed among U.S. adult populations with the full Household Food Security Survey Module and accordingly our findings may have been weakened by measurement error associated with including only a small portion of items from this tool on the EAT 2018 survey^(33)^. The adolescent survey at baseline included different measures of food insecurity that were drawn from a validated tool and similar to measures included in the Child Food Security Survey Module, but were not validated for use in young people this age^(41)^. Both measures of food insecurity were focused on food access problems, and thus, it is possible the assessment underestimated food insecurity and our study accordingly underestimated the true association between ACE and food insecurity stemming from challenges unrelated to food access. Retrospective self-reports of ACE may suffer from recall bias; however, retrospective recall is often the only feasible way to assess abuse and neglect in large epidemiologic cohorts. Further, it is not clear the extent to which retrospective report is less accurate than prospective assessments, which also suffer from underreporting and measurement error. Second, there was substantial attrition of the sample over longitudinal follow-up. If attrition is correlated with both ACE and food insecurity, then our findings may be affected by selection bias. IPW was designed to reduce this bias by weighting the sample to represent the original baseline, population-based sample.

There are several implications of the current study findings within the context of the growing evidence for developing trauma-informed policies and services to prevent food insecurity and the many adversities that young people have experienced during the COVID-19 pandemic. Primary prevention efforts to protect young people from ACE are of paramount importance along with efforts to address the potential consequences of adversity for outcomes that impact access to adequate food (e.g. low wages). For example, interventions designed to support young people with ACE to improve their academic performance and job skills could help them to earn adequate money for food. The results described here emphasise the great significance of providing supports for young people navigating the transition to adulthood during and in the aftermath of the COVID-19 pandemic. There is some evidence that young people with a history of ACE have experienced increased risk for financial difficulties during the COVID-19 pandemic^(42)^. Health care providers and community-based nutrition professionals are well-positioned to collaborate with social work professionals to screen emerging adults for experiences of adversity so that supports can be provided for accessing food assistance, training programs, and mental health services; accordingly there are several opportunities to improve the implementation of best practices for providing referrals. It is noteworthy that emotional abuse was the most consistent and strongest risk factor for food insecurity in our study, yet this form of ACE is often hidden. A history of experiencing this ACE may not be recognised unless sensitive screening is implemented by health care providers. In the next phases and aftermath of the COVID-19 pandemic, it will be particularly important for health care providers to attend to abuse and other ACE (e.g. coercive control, mental health challenges resulting from stress associated with the pandemic) that may have increased or been hidden as a result of stay-at-home orders and societal trends in virtual work and school arrangements^(43)^. Importantly, any screening that occurs must be accompanied by evidence-based strategies for how to support individuals who reveal an ACE history. For example, there may be opportunities for health care providers to support young people with ACE histories by teaching positive coping strategies, identifying risks for depression and other mental health problems and/or making referrals to supports and resources (mental healthcare providers, safety net programs) that can reduce the risk for food insecurity.

The current study also extends evidence in support of implementing trauma-informed policies and program models to provide food assistance for young people who have experienced adversity. A framework for developing trauma-informed policy has been developed and its core principles can be used to guide future efforts to address food insecurity^(44)^. Many innovative policy responses to food insecurity were implemented during the COVID-19 pandemic, and there is now an opportunity for health care providers and community-based nutrition professionals to advocate for the continuation of effective policies. Temporary policies of relevance to emerging adult populations provided for an increase in Supplemental Nutrition Assistance Program (SNAP) benefit levels; expansion of eligibility for SNAP as a result of suspending the time limit for able-bodied adults without dependents; waiving or extending paperwork deadlines and interview requirements for SNAP; expansion of the fruit and vegetable benefit allotted by the Special Supplemental Nutrition Program for Women, Infants, and Children; the flexibility for SNAP benefits to be redeemed online and the creation of the Farmers to Families Food Box Program^(45)^. There is some evidence that these program modifications worked synergistically to prevent a steep rise in food insecurity during the pandemic^(46)^; however, as these temporary policies expire it is timely to attend to the many barriers that emerging adults experience in accessing food assistance and programmatic strategies for addressing safety, trustworthiness and transparency, collaboration, empowerment and choice^(44)^. To improve on existing policies, it could be important to address barriers to eligibility for food assistance benefits and attend to the stigma associated with accessing various forms of assistance. For example, most emerging adults who are enrolled in college or university studies are not currently eligible to receive SNAP benefits,^(47)^ and the modification of eligibility requirements could be of great benefit to young people who are successfully admitted to a postsecondary institution. There is also a great need for the development of other policies to reach populations outside of college and university settings. Emerging adults further report experiencing stigma when accessing emergency food resources and therefore may particularly benefit from trauma-informed programs that attend to the principles of ensuring safety, empowerment, choice and collaboration (e.g. inviting recipients to assist with cooking and serving)^(48)^.

In summary, the results of this study indicate that food insecurity is more prevalent among young people who have a history of ACE. Additional research addressing how these types of traumatic and developmentally disruptive experiences may inhibit consistent access to the safe, healthy and affordable foods that are essential for optimal health and well-being would be beneficial to better guide interventions. It will be particularly important for future studies to comprehensively investigate linkages between ACE and markers of nutrition security. Nutrition security is a broader concept than food security and addresses having equitable and stable availability, access, affordability and utilisation of foods and beverages that promote well-being and prevent and treat disease^(49)^. Research addressing nutrition security is critical to build understanding of barriers relating to consumption of a nutritionally adequate diet. Measures of nutrition security have recently been developed and validated in U.S. samples^(50)^, and therefore it will be feasible for future research to explore this construct of relevance to address the elevated rates of overweight, obesity and chronic disease among persons with a history of ACE^(3,5)^. To directly inform the development of trauma-informed programming, studies could also attend to whether persons who have experienced ACE are able to access food assistance when they need it or whether an ACE history also impedes successful navigation of our complex safety net system. It will be imperative for future studies to more comprehensively assess food insecurity by attending to problems other than those related to food access (e.g. problems relating to food management, difficulty in accessing culturally familiar foods).

## Supplementary material

For supplementary material accompanying this paper visit https://doi.org/10.1017/S1368980023001349. click here to view supplementary material
