# Supplementary material for: Adverse childhood experiences and food insecurity in emerging adulthood: findings from the EAT 2010–2018 study
Source: Public Health Nutr. 2023 Jul 11;26(11):2343–54. doi: 10.1017/S1368980023001349 (PMC10641614; doi:10.1017/S1368980023001349)
Supplement: Supplementary file 1 [file S1368980023001349sup001.docx]

| **Supplementary Table 1.** Sample characteristics: emerging adults (N=1,518) who participated at baseline in the EAT 2010 study in Minneapolis-St. Paul, Minnesota and reported on food insecurity (food secure n=1,178; food insecure n=340) as part of a follow-up survey in 2017-2018^*^ | | | | |
| --- | --- | --- | --- | --- |
| Characteristics | Overall sample, n | Overall sample, %^†^ | Food secure sample, %^†^ | Food insecure sample, %^†^ |
| Gender |  |  |  |  |
| Female | 878 | 53.0 | 51.7 | 57.1 |
| Male | 629 | 46.4 | 47.8 | 41.9 |
| Other gender identity | 11 | 0.6 | 0.5 | 1.1 |
| Ethnicity/race |  |  |  |  |
| White | 359 | 19.1 | 20.3 | 15.2 |
| Black or African American | 330 | 28.8 | 26.9 | 35.1 |
| Hispanic or Latino | 263 | 16.8 | 18.1 | 12.3 |
| Asian American | 346 | 20.1 | 21.3 | 16.3 |
| Native American | 59 | 3.6 | 3.3 | 4.5 |
| Mixed or other | 156 | 11.6 | 10.1 | 16.7 |
| Household socioeconomic status (SES)^ǂ^ |  |  |  |  |
| Low | 539 | 38.9 | 36.1 | 48.2 |
| Low-middle to middle | 576 | 40.4 | 41.7 | 35.9 |
| Upper-middle to high | 366 | 20.7 | 22.2 | 15.9 |
| Student status among those with no  postsecondary degree/certificate |  |  |  |  |
| Not a student | 597 | 55.9 | 53.0 | 64.8 |
| High school student | 45 | 4.6 | 4.7 | 4.0 |
| Postsecondary student | 470 | 39.5 | 42.3 | 31.2 |
| Educational attainment among nonstudents |  |  |  |  |
| No high school degree | 73 | 9.4 | 7.0 | 16.0 |
| High school graduate or equivalent | 524 | 65.6 | 65.5 | 66.0 |
| Associate or trade degree | 86 | 10.3 | 9.9 | 11.1 |
| 4-year college or advanced degree | 130 | 14.7 | 17.6 | 6.9 |
| Employment status |  |  |  |  |
| Working full-time | 771 | 51.7 | 51.3 | 52.8 |
| Working part-time | 422 | 27.4 | 28.0 | 25.5 |
| Stay at home caregiver | 47 | 3.1 | 3.2 | 2.8 |
| Unemployed, seeking work | 171 | 11.8 | 10.7 | 15.4 |
| Not working for pay | 94 | 6.0 | 6.8 | 3.4 |
| Live with a parent | 731 | 48.7 | 51.9 | 38.2 |
| Live with a child(ren) of your own | 150 | 9.9 | 7.9 | 16.3 |
| Food insecure during adolescence | 182 | 12.3 | 10.0 | 20.1 |
| Adverse childhood experiences (ACE history reported) |  |  |  |  |
| Sexual abuse | 226 | 14.0 | 11.6 | 21.9 |
| Physical abuse | 245 | 16.9 | 13.2 | 29.1 |
| Emotional abuse | 191 | 12.5 | 8.3 | 26.3 |
| Incarceration of household member | 165 | 11.4 | 8.1 | 22.3 |
| Substance abuse by household member | 330 | 22.1 | 17.3 | 38.0 |
| Mental health problem of household  member | 404 | 25.9 | 21.1 | 41.5 |

^*^Food security status was defined by responses to the question “In the last 12 months, did you ever eat less than you felt you should because there wasn’t enough money for food?” and “In the last 12 months, were you ever hungry but didn’t eat because there was not enough money for food?”. Food insecurity was determined by reporting yes to both questions.

^†^Values are weighted to reflect the probability of responding to the follow-up EAT 2018 survey.

^ǂ^The primary determinant of SES was parental educational level, defined by the higher level of either parent. Additional measures of income and employment were used as part of an algorithm to reduce the impact of missing data and to prevent misclassification in ranking SES (range: 1-5). Low SES was defined as rank 1, middle SES as rank 2-3, and high SES as rank 4-5.

Figure Legend

**Figure 1.** Conceptual model guiding analysis of the associations between adverse childhood experiences, childhood socioeconomic status, and food insecurity in emerging adulthood among participants in the EAT 2010-2018 (Eating and Activity over Time) longitudinal study.
